# The relationship between social responsibility fulfilment and industry competitiveness: Empirical evidence from construction industry in China

**DOI:** 10.1371/journal.pone.0295181

**Published:** 2023-11-30

**Authors:** Huan Shu, Jingyi Duan

**Affiliations:** School of Business, Hohai University, Nanjing, China; Yunnan Technology and Business University, CHINA

## Abstract

Social responsibility fulfilment is an important part of the operation management (OM) and competitiveness of the construction industry (CIC). This study investigates the mechanisms through which social responsibility fulfilment in the construction industry affects industry competitiveness and the mediating role of industry operations management. From the intra-organizational stakeholder perspective, social responsibility fulfilment (SR) is divided into two dimensions: shareholder-dimensional (SH) and employee-dimensional social responsibility fulfilment (EM). By analysing a panel of the construction industry in 12 provinces of China from 2015 to 2020, an inverted U-shaped relationship between social responsibility fulfilment and construction industry competitiveness and between shareholder-dimensional social responsibility fulfilment and construction industry competitiveness was found, while a U-shaped relationship between employee-dimensional social responsibility fulfilment and construction industry competitiveness was found. Furthermore, social responsibility fulfilment and shareholder-dimensional social responsibility fulfilment had significant effects on operation management. Additionally, the study concludes that operation management is positively related to construction industry competitiveness and plays a partially mediating role between social responsibility fulfilment and construction industry competitiveness. The findings enrich the theoretical basis of the relationship between social responsibility fulfilment and competitiveness and demonstrates important management significance for the construction industry in managing its social responsibility behaviour and improving industry competitiveness, thus promoting high-quality development of the construction industry.

## 1. Introduction

The construction industry holds a pivotal position in the economic development of numerous countries [1], including developing countries such as China [2]. China’s construction industry has been identified as a key contributor to the country’s national economy and has improved its Gross National Product over time [3]. According to the National Bureau of Statistics of China, the total value of China’s construction enterprises will reach approximately US$4.3 trillion by 2021. In addition, with the development of economic globalization, China’s construction industry has gradually moved outside its own borders and has launched engineering and construction activities in over 190 countries and regions worldwide. Its number of enterprises and business share have ranked first in the world for more than a decade (Ministry of Housing and Urban-Rural Development, 2021), demonstrating its comprehensive strength [4]. Currently, China’s construction industry has a market share of approximately 21.53% of the global construction market (Engineering News-Record, 2021) and plays a crucial role in global infrastructure construction and the development of the construction industry.

With intensifying industry competition, significant deficiencies in social responsibility fulfilment have been exposed, leading to several unresolved issues [4, 5]. For example, quality and safety accidents occur frequently in construction engineering [6], and employees’ rights and interests are not guaranteed [7]. These phenomena have been studied extensively by industry stakeholders and scholars. Social responsibility is a deliberate corporate effort to improve society and maintain sustainable development [8–12], and can effectively assist industries in improving their competitiveness while avoiding the above problems [13]. Thus, social responsibility is gradually being emphasised and fulfilled in the construction industry. Managers have gradually realised that focusing only on short-term financial goals is insufficient [14] and have recognised the significant impact of social responsibility fulfilment on the industry’s competitiveness [15].

Some scholars have investigated social responsibility fulfilment (SR) as a potential means of enhancing competitive advantage at the macro level [16]. With the intensification of global market competition, the construction industry can employ social responsibility as a viable strategy to enhance its image, which helps to secure competitiveness and foster high-quality advancement [17]. However, the impact of social responsibility fulfilment on competitiveness remains unclear. Thus, it may be worthwhile to investigate the potential mechanisms that influence the relationship between social responsibility fulfilment and industry competitiveness at the micro level. Moreover, previous research on social responsibility has focused primarily on its external dimension, ignoring research from the internal perspective of the organisation; in particular, research on employee-dimensional social responsibility fulfilment (EM) is still relatively lacking [18]. Thus, this study investigates the following questions: Is there a relationship between the two dimensions of social responsibility fulfilment, shareholders and employees, as the two core stakeholders within the organisation, and competitiveness? Can the fulfilment of a single dimension of social responsibility impact competitiveness? Furthermore, most studies have been conducted in the context of developed countries in Europe and the United States, with few conducted in the context of developing countries, especially representative emerging economies [19]. Compared to developed countries, firms in developing countries may place more emphasis on operation management (OM) goals [20], thus social responsibility fulfilment may differ, and the relationship between social responsibility fulfilment and competitiveness may differ. In particular, in a social environment with Chinese characteristics, corporate social responsibility behaviours are influenced by government behaviour and policies [21]. In this case, it is unclear whether social responsibility fulfilment indirectly affects competitiveness by affecting the level of OM.

Motivated by the challenge of addressing the limitations of existing studies, this study considers China, a rapidly growing emerging economy, as its main research object. Based on the internal perspective of an organisation, we focus on the construction industry and introduce OM as a mediating variable to investigate the influential mechanisms of social responsibility fulfilment and construction industry competitiveness (CIC). This study makes the following contributions.

(1) This study finds that the fulfilment of social responsibility in China’s construction industry has limitations in terms of improving competitiveness and OM. Only when the input of social responsibility is controlled within a reasonable range can it take full advantage of its effectiveness. The research results can help organisations position social responsibility strategically and adjust social responsibility input to the best level through effective resource allocation to maximise the effectiveness of the social welfare supply.

(2) This study reveals the intermediary role of operations management in the non-linear transformation path of the two, which provides a new path for managers to formulate a coordinated development strategy of social responsibility and sustainable competitiveness and establish a long-term governance mechanism for social responsibility in the construction industry.

(3) Based on the existing research, this study further clarifies the differentiated impact mechanism between the different dimensions of social responsibility fulfilment (shareholders and employees), industry competitiveness, and operations management. The research results not only enrich the existing theories but also put forward targeted strategic suggestions for construction enterprises to promote the diversification, balance, and coordination of social responsibility, which is helpful in promoting the high-quality transformation of the construction industry.

The remainder of this paper is organised as follows. Section 2 reviews the literature and presents our hypotheses. Section 3 describes the data, variables, and models used in our empirical analysis. Section 4 presents the empirical analysis and tests the hypotheses. Section 5 presents the results. Finally, Section 6 presents the conclusion and reflects on the limitations of the study.

## 2. Literature review and research hypotheses

### 2.1 Social responsibility and competitiveness

Academic research in the realm of social responsibility empirical analysis has always focused on the relationship between social responsibility and competitiveness. Many scholars hold that a positive linear correlation exists between the two [22, 23]. According to the ‘social impact hypothesis’ proposed by Cornell and Shapiro [24], proactive social responsibility fulfilment by an enterprise can elicit positive responses from stakeholders, which ultimately contribute to its sustainable development [22]. According to stakeholder theory, social responsibility fulfilment can meet the deep-seated needs of employees for happiness, value, and belonging in the organisation [25], which in turn helps enhance productivity and innovation [26]. Moreover, social responsibility fulfilment can increase organizational reputation and industry profits, safeguard the rights and interests of shareholders, and ensure the industry’s financial stability and adequacy for development [27].

Social responsibility fulfilment can bring competitive advantages to organizations. Social responsibility activities in the construction industry can lead to favourable resource allocation for stakeholders and enhance the competitive advantage of organisations through an improved reputation and image [28]. However, these resources and positive responses are limited in quantity and type [29]. In other words, organisations cannot provide unlimited beneficial resources for all stakeholders, and stakeholders are unlikely to provide unlimited positive responses to the organisation. Thus, excessive social responsibility fulfilment may affect normal operational activities and negatively affect industry competitiveness [30]. For example, shareholders are an important source of organizational funds, and shareholder-dimensional social responsibility fulfilment is equivalent to an investment that can accumulate more social capital for the organisation to support the development of operational activities [28]. However, excessive shareholder-dimensional social responsibility fulfilment is counterproductive and not conducive to improving competitiveness. In addition, the fulfilment of employee-dimensional social responsibilities, such as improving the working environment and ensuring the health of employees, is crucial to the sustainable development of organisations [31], but it is a long-term accumulation process [32]. In the early stages of employee-dimensional social responsibility fulfilment, scarce resources, such as funds, facilities, human resources and other necessary resources, will be occupied to enhance competitiveness. The organisation may occupy resources but fail to make reasonable use of potential benefits [33], thus negatively impacting competitiveness. When employee-dimensional social responsibility fulfilment reaches a mature level, potential benefits are transformed into tangible results, such as forming a good reputation, to enhance the organisation’s attractiveness to potential employees and obtain industrial competitive advantages [34]. The intangible capabilities of these employees, such as intellectual capital, financial knowledge, and business experience, can help organisations acquire unique external resources, which can be transformed into competitiveness [35].

Based on the above theoretical analysis, this study argues that there may not be a simple linear relationship between social responsibility fulfilment and construction industry competitiveness; instead, it is a more complex non-linear relationship. As core stakeholders within an organisation, shareholders and employees may also have different non-linear effects on competitiveness. Therefore, based on the non-linear perspective, this study proposes the following hypotheses:

#### Hypothesis 1

The relationship between social responsibility fulfilment and the competitiveness of the construction industry is a non-linear relationship.

#### Hypothesis 1a

The relationship between shareholder-dimensional social responsibility fulfilment and competitiveness in the construction industry satisfies a non-linear relationship.

#### Hypothesis 1b

The relationship between employee-dimensional social responsibility fulfilment and construction industry competitiveness satisfies a non-linear relationship.

### 2.2 Social responsibility and operation management

Operative management has long been a focus of attention in the realm of management science. Social responsibility fulfilment, as a win-win business mode of cooperation between enterprises and stakeholders, will certainly have an impact on the enterprises’ operational activities. ‘ISO26000: Guidance on Social Responsibility’ mentioned that social responsibility fulfilment will have a positive impact on many aspects of enterprises’ operation management. Active social responsibility fulfilment is conducive to attracting employees, retaining quality customers, and strengthening relationships between enterprises and investors, suppliers, and other important stakeholders [36]. In addition, social responsibility fulfilment will maintain good communication between an enterprise and a wide range of stakeholders, such as the government and the community, and help the enterprise establish a good external image, which is conducive to the development of production and operational activities, thus helping the enterprise improve its management level [37].

Academic circles have confirmed the positive effect of social responsibility fulfilment on enterprise operations management. Gregory et al. [38] proved that social responsibility fulfilment can effectively reduce the operational risks of an enterprise and enhance its operational capacity, thus promoting the sustainable development of operation management. Irene et al. [39] emphasised the importance of social responsibility in the sustainable development of an enterprise’s operational activities, with higher levels of social responsibility leading to an improved image and operational ability, which results in higher returns. Chen et al. [40] argue that internal organizational social responsibility fulfilment reduces employee turnover, enhances employee loyalty, and improves enterprise reputation, which, in turn, improves corporate operations management. Based on the theory of signal transmission, the behaviour of a construction enterprise in fulfilling its social responsibility can convey a trustworthy positive signal to the outside world. This is conducive to the establishing a good relationship with stakeholders to obtain resources and a good operational environment [41], thus creating more sustainable value for the enterprise and improving its operation management [42].

Based on the theory of limited resources, some scholars, such as Lin et al., believe that the benefits that stakeholders can provide to enterprises are limited and the benefits an enterprise can derive from social responsibility are finite [43]. Therefore, linear growth of OM through social responsibility fulfilment is unrealistic. Overly ambitious social responsibility activities consume more internal resources, and their income cannot make up for the cost. Thus, enterprise operation management experiences negative effects, which is not conducive its sustainable development [44]. In the construction industry, in particular, fulfilling social responsibility through safety education and employee training can incur high costs. Therefore, excessive social responsibility activities are not conducive to improving an enterprise’s OM.

Based on this theoretical background, this study argues that social responsibility fulfilment does not have a linear positive impact on enterprise operations management, and there may be a complex non-linear relationship between them. Therefore, this study proposes the following hypotheses from the perspective of stakeholders within an organisation:

#### Hypothesis 2

The relationship between social responsibility fulfilment and enterprise operation management is a non-linear relationship in the construction industry.

#### Hypothesis 2a

The relationship between shareholder-dimensional social responsibility fulfilment and enterprise operations management is a non-linear relationship in the construction industry.

#### Hypothesis 2b

The relationship between employee-dimensional social responsibility fulfilment and enterprise operations management satisfies a non-linear relationship in the construction industry.

### 2.3 Operation management and industry competitiveness

Operational management is one of the most active branches of the management sciences. Operations management can identify and eliminate behaviours and resources that are not conducive to the improvement of enterprise efficiency and optimise the operation process to increase its value and improve organizational efficiency and productivity. Many experts and scholars have realised the close relationship between operations management and competitiveness and believe that operations management is the fundamental source of competitiveness. Kholopane [45] considered that good OM can help enterprises meet market demands and compete for market share by providing high-quality services and products. Thus, these enterprises can obtain competitive advantages that differ from those of other enterprises. Okwang’a et al. [46] proposed that good operational and management strategies can help enterprises efficiently use organizational resources and improve stakeholder satisfaction with limited resources, helping them maintain a leading position in market competition. Mantje et al. [47] found that effective OM strategies can create a good operating environment for enterprises and help maintain communication between enterprises and external stakeholders. An excellent operating environment is necessary to enhance an enterprise’s resource integration and utilisation ability and improve its profitability. Therefore, effective OM strategies are helpful for enterprises to gain market competitive advantages.

Studies show that possessing an excellent operation management strategy is a significant factor for improving competitiveness. Construction enterprises not only maximise their own value through a differentiation strategy but also obtain a good market share in effective operational activities, which will help improve the market share of the construction industry and form an industrial competitive advantage that is different from other industries. The strong OM of construction enterprises indicates that their strategic management and financial structure arrangements are sound. It not only provides a strong financial guarantee for sustainable development but also enhances anti-risk strength to improve core competitiveness [48]. However, according to information transfer theory, better enterprise operation management conveys positive development potential information to the external world, which can enhance stakeholder interest [49]. Thus, it can enhance an enterprise’s prestige and create a differentiated competitive advantage. Good operations management is crucial to enhance competitiveness [50]. Especially in construction industry, the higher the degree of recognition of the owner, the higher is the probability of winning the bid, which helps enterprises obtain more production and management opportunities [51]. Effective production and operational activities not only improve the profitability of enterprises but also improve the overall value of the construction industry. The construction industry occupies a large market share to enhance its competitive advantage.

Based on the theoretical and logical deductions of the above research, the following hypotheses are proposed:

**Hypothesis 3.** Operational management in the construction industry is significantly positively correlated with industry competitiveness.

### 2.4 The intermediary effect of industry operation management

This study speculates that OM plays an intermediary role in the relationship between social responsibility fulfilment and competitiveness in the construction industry. Specifically, when the level of social responsibility fulfilment in the construction industry is low, it indicates that enterprises in the industry still lack social responsibility implementation [52], which may lead to negative emotions among stakeholders towards enterprises [53]. For example, the absence of social responsibility fulfilment damages trust between enterprises, shareholders, and potential investors, affecting the normal development of investment and financing activities of enterprises and, therefore, negatively impacting corporate operations [54]. When employees perceive that the enterprise is stingy and their rights and interests are not protected, their work enthusiasm and efficiency decline, which is not conducive to the improvement of operation and production efficiency [55]. However, enterprise operation management is important for enterprise survival and profitability, and high OM performance is a key driving factor for improving enterprise competitiveness [45]. Therefore, the lack of social responsibility fulfilment will negatively impact the improvement of the OM level, which is not conducive to improving competitiveness, as well as the construction industry’s ability to occupy a favourable market share; thus hindering the formation of competitive advantages. The improvement of social responsibility fulfilment in the construction industry also means an enhancement of corporate social responsibility fulfilment in the industry. Therefore, stakeholders’ perceptions of construction enterprises will improve, and it will be easier for enterprises to obtain stakeholders’ support and key resources controlled by them [41]. Once this occurs, enterprises and stakeholders will form positive interactions, which will aid in the smooth development of enterprise OM activities [54]. Construction enterprises can improve their OM abilities and enhance their competitive advantage through high operational performance. This can help the construction industry obtain a considerable market share and effectively promote industry competitiveness.

However, excessive social responsibility fulfilment by construction enterprises does not improve OM. With increasing investment in social responsibility, its marginal effect will eventually decline [29], triggering a negative impact on operations management and weakening its competitive advantage. On the one hand, excessive social responsibility activities of construction enterprises may incur significant additional costs, and given the limited resources provided by stakeholders, the benefits are unable to cover the costs [29]. On the other hand, excessive social responsibility fulfilment may convey the existence of idle resources to stakeholders [56], trigger a negative perception of poor operation to enterprises, and thus reduce stakeholders’ cooperative intent and hamper the development of normal operational activities. Therefore, excessive social responsibility fulfilment will have unfavourable effects on the improvement of enterprise operation management levels, which will not only hinder enterprise competitiveness improvement but also negatively impact on the acquisition of industrial competitive advantages.

Based on the theoretical analysis, this study proposes the following hypotheses.

#### Hypothesis 4

Operational management mediates the relationship between social responsibility fulfilment and competitiveness in the construction industry.

#### Hypothesis 4a

Operational management plays a mediating role between shareholder-dimensional social responsibility fulfilment and competitiveness in the construction industry.

#### Hypothesis 4b

Operational management plays a mediating role between employee-dimensional social responsibility fulfilment and the competitiveness of the construction industry.

## 3 Data, variables, and models

### 3.1 Sample and data

This study focuses on the association between social responsibility fulfilment, industry competitiveness, and industry operations management using a sample of listed Chinese construction companies from 2015 to 2020. To ensure the robustness of the data, we exclude ST- and PT-type companies as well as those with significant data deficiencies. The sample companies were from 12 provinces: (1) the eastern region: Beijing, Shanghai, Jiangsu, Zhejiang, Shandong, and Guangdong; (2) the central region: Heilongjiang, Anhui, and Hubei; and (3) the western region: Chongqing, Sichuan, and Xinjiang. The selected companies reflect the industry’s development level. Therefore, we use the average social responsibility fulfilment level of these construction enterprises to represent the social responsibility fulfilment levels of these provinces and cities. To accurately measure the competitiveness of the construction industry in these provinces, a construction industry competitiveness evaluation index system was established, and the original data for each index were obtained from data published by the China Statistical Yearbook, China Construction Industry Statistical Yearbook, China Construction Industry Yearbook, and the National Bureau of Statistics. Other financial data on the construction industry are obtained from the China Stock Market and Accounting Research Database (CSMAR). In this study, the data were analysed and processed using EXCEL, SPSS, and STATA software and winsorised (1%-99%) to avoid the influence of outliers.

### 3.2 Variables

#### Social responsibility

Currently, there is a lack of authoritative institutions that quantitatively measure social responsibility fulfilment levels of the construction industry in various provinces and regions in China. Therefore, this study utilises the method employed in the ‘Research Report on Corporate Social Responsibility of China’ (2021) to calculate the average social responsibility score of listed construction enterprises in 12 provinces to represent the level of social responsibility fulfilment in the construction industry in these regions. The social responsibility of each company and the two specific dimensions of CSR data of shareholders and employees were measured based on data from the Hexun database of the social responsibility of listed companies. This database is widely used in empirical CSR research and is currently recognised as the most comprehensive and authoritative CSR database in China.

#### Construction industry competitiveness

Currently, the academic community has not reached a consensus on the evaluation index system for the CIC. To comprehensively measure China’s construction industry competitiveness, this study adopts the ‘Diamond Model’ proposed by Michael Porter [57] and refers to existing research results in China. Eighteen indicators were selected from four dimensions: production factors and resources, development and support conditions, industry efficiency and structure, and related and supporting industries. These indicators were evaluated at four levels, including the target system, subsystem, criterion, and indicator levels, to build an evaluation index system for the CIC, as shown in Table 1.

**Table 1:** Construction industry competitiveness evaluation index system.

Target layer	Subsystem layer	Guideline layer	Indicator layer	Unit
Construction Industry Competitiveness	Production factor resources	Capital Resources	fixed assets (*x*_1_)	million yuan
			corporate liabilities (*x*_2_)	million yuan
		Human Resources	practitioners (*x*_3_)	person
		Infrastructure	technical equipment rate (*x*_4_)	yuan per person
			power equipment rate (*x*_5_)	kilowatt per person
	Development and support conditions	Development Conditions	total output (*x*_6_)	billion
			construction industry increase (*x*_7_)	pcs
			number of business units (*x*_8_)	billion
		Support Conditions	real estate development investment (*x*_9_)	billion
	Industry efficiency and structure	Industry Benefits	total corporate revenue (*x*_10_)	million yuan
			total corporate profit (*x*_11_)	million yuan
			total corporate tax (*x*_12_)	million yuan
			labor productivity in terms of GDP (*x*_13_)	yuan per person
		Structure Level	the proportion of non-publicly owned enterprises in the construction industry (*x*_14_)	%
	Related industries and supporting industries	Auxiliary Industries	number of survey and design institutions units (*x*_15_)	pcs
			number of construction supervision enterprise units (*x*_16_)	pcs
			number of engineering bidding agency units (*x*_17_)	pcs
		Support Industries	number of real estate business units (*x*_18_)	pcs

To accurately evaluate the competitiveness of China’s construction industry in the twelve provinces from 2015 to 2020, this study utilised the evaluation index system presented in Table 1. The original data for each indicator were obtained from authoritative sources such as the ‘China Statistical Yearbook’, ‘China Construction Industry Statistical Yearbook’, ‘China Construction Industry Yearbook’, and ‘National Bureau of Statistics’. Given that this study focuses on the dynamic evaluation of competitiveness, panel data were selected, and a time-series global principal component analysis (PCA) was conducted to evaluate the competitiveness of the construction industry in each province.

In this study, SPSS software was used for the PCA, and Kaiser-Meyer-Olkin measurement (KMO) and Bartlett’s test of sphericity were first performed to verify whether the selected index system could be subjected to a time-series global PCA. A detailed overview of the test outcomes is presented in Table 2.

**Table 2:** Results of KMO and Bartlett’s test of sphericity.

KMO Sampling suitability quantity		0.795
Bartlett’s test of sphericity	Approximate cardinality	2619.717
	Degree of freedom	153
	Significance	0.000

Based on the information presented in Table 2, the suitability of the selected index system for time-series global PCA was examined using the KMO and Bartlett’s test of sphericity. The value of KMO was 0.795 (greater than 0.6), and the significance of Bartlett’s test was 0.000 (less than 0.05), which indicated that the approximate chi-square value of Bartlett’s test was large, and the sample size was sufficient for analysis. Therefore, the selected 18 variables were suitable for the time-series global PCA. Next, a PCA was conducted to determine the eigenvalues, contribution of eigenvalues, and cumulative contribution of the correlation coefficient matrix, as presented in Table 3.

**Table 3:** Eigenvalues, eigenvalue contribution, and cumulative contribution of the correlation coefficient matrix.

Ingredients	Initial Eigenvalue			Extraction of the sum of squares of loads		
	Total	Variance%	Accumulation %	Total	Variance%	Accumulation%
1	10.662	59.235	59.235	10.662	59.235	59.235
2	2.822	15.678	74.913	2.822	15.678	74.913
3	1.771	9.841	84.755	1.771	9.841	84.755
4	0.941	5.228	89.983			
5	0.443	2.460	92.443			
6	0.401	2.227	94.670			
7	0.242	1.344	96.014			
8	0.188	1.044	97.058			
9	0.159	0.885	97.943			
10	0.146	0.808	98.751			
11	0.107	0.592	99.344			
12	0.051	0.282	99.626			
13	0.030	0.168	99.794			
14	0.024	0.131	99.925			
15	0.006	0.035	99.959			
16	0.004	0.023	99.983			
17	0.002	0.012	99.995			
18	0.001	0.005	100.000			

Note. The extraction method is the PCA.

Based on Table 3, it is evident that the first three factors are public factors that can be derived through PCA. The cumulative contributions of the three factors, namely *F*_1_, *F*_2_, and *F*_3_, amounted to 84.755%, with individual contributions of 59.235%, 15.678%, and 9.841%, respectively. This indicates that the first three factors correspond to most of the information in the original variables. Therefore, the first three factors were extracted factors as the primary factors. To obtain common factor loadings, the eigenvectors corresponding to the eigenvalues of the three common factors were used to calculate the to-rotation factor load matrices, as shown in Table 4.

**Table 4:** Rotated factor loadings matrix of competitiveness indicators of sample industries.

	Ingredients		
	1	2	3
*x*_1_	0.778	0.565	-0.162
*x*_2_	0.715	0.623	-0.141
*x*_3_	0.827	-0.441	-0.114
*x*_4_	-0.105	0.859	0.145
*x*_5_	-0.074	0.528	0.693
*x*_6_	0.929	-0.133	-0.196
*x*_7_	0.866	-0.279	0.217
*x*_8_	0.942	-0.229	0.136
*x*_9_	0.877	-0.113	0.127
*x*_10_	0.955	0.090	-0.202
*x*_11_	0.862	0.235	-0.223
*x*_12_	0.907	0.049	-0.197
*x*_13_	0.122	0.703	-0.356
*x*_14_	0.562	-0.207	-0.571
*x*_15_	0.836	0.294	0.158
*x*_16_	0.913	-0.059	0.251
*x*_17_	0.756	-0.027	0.525
*x*_18_	0.813	-0.225	0.381

After extracting the principal component factors, the principal component score coefficient matrix, which serves as a representation of the original variables, was computed using SPSS software. The results are summarised in Table 5.

**Table 5:** Matrix of component score coefficients of competitiveness indicators of sample industries.

	Ingredients		
	1	2	3
*x*_1_	0.073	0.200	-0.092
***x*_2_**	0.067	0.221	-0.079
*x*_3_	0.078	-0.156	-0.064
*x*_4_	-0.010	0.304	0.082
*x*_5_	-0.007	0.187	0.391
*x*_6_	0.087	-0.047	-0.111
*x*_7_	0.081	-0.099	0.123
*x*_8_	0.088	-0.081	0.077
*x*_9_	0.082	-0.040	0.072
*x*_10_	0.090	0.032	-0.114
*x*_11_	0.081	0.083	-0.126
*x*_12_	0.085	0.017	-0.111
*x*_13_	0.011	0.249	-0.201
*x*_14_	0.053	-0.073	-0.322
*x*_15_	0.078	0.104	0.089
*x*_16_	0.086	-0.021	0.142
*x*_17_	0.071	-0.010	0.296
*x*_18_	0.076	-0.080	0.215

This study employed PCA to extract three common factors from the samples and obtained the factor scores of each sample by calculating the score coefficient matrix of each factor. The weights were then determined based on the cumulative proportion of the variance contribution of each principal component. This process results in a comprehensive score function for construction industry competitiveness, which is represented by the following formula:

$$F=(0.5924*F_{1}+0.1568*F_{2}+0.0984*F_{3})/0.8476.$$

#### Operation management

Operating performance is an important indicator for reflecting the level of an enterprise’s OM. Existing research commonly uses indicators to measure operating management performance, including financial indicators, such as return on equity, return on assets, and return on earnings per share. Market indicators, such as Tobin’s Q. These two methods have their own advantages and disadvantages; however, financial indicators have strong stability and can accurately reflect the operational situation. This study measures the levels of industry operations management indicators for the construction sector in each region by calculating the average return on assets (ROA) of all listed enterprises within the local construction industry. The average return on equity (ROE) was used in the robustness test.

#### Control variables

This study utilises a set of control variables to investigate their influence on the focal variables. The selected control variables include economic development (ECO), level of infrastructure development (INF), level of industrial development (IND), level of construction development (CON), level of financial sector development (FIN), level of openness to the outside world (OPEN), level of government intervention (GOV), population density (PD), province (PRO), and year (YEAR). Table 6 displays all the variables examined in this study.

**Table 6:** Variable design and definition.

	Variable Name	Variable Symbols	Variable Definition
Dependent variable	Construction Industry Competitiveness	*CIC*	PCA yielded
Independent variable	Social-Responsibility Fulfilment	*SR*	Hexun CSR Rating Average
	Shareholder-Dimensional Social Responsibility Fulfilment	*SH*	Hexun Social Responsibility Index Shareholder Dimension Rating Average
	Employee Dimensional Social Responsibility Fulfilment	*EM*	Hexun Social Responsibility Index Employee Dimension Rating Average
	Operation Management	*OM*	The return on assets of listed companies in the construction industry by province is taken as the average value
Control variables	Degree of Economic Development	*ECO*	GDP per capita taken as logarithm
	Infrastructure Development Level	*INF*	Growth rate of fixed asset investment
	Industrial Development Level	*IND*	Industrial Value Added/GDP
	Construction Industry Development Level	*CON*	Value Added of Construction Industry/GDP
	Financial Industry Development Level	*FIN*	Financial Sector Value Added/GDP
	Level of External Opening	*OPEN*	Amount of Foreign Direct Investment/GDP
	Level of Government Intervention	*GOV*	Fiscal Spending by Province/GDP
	Population Density	*PD*	(Urban Population + Urban Transient Population)/Urban area
	Province	*PRO*	Individual firm dummy variables, controlling for individual firm fixed effects
	Year	*YEAR*	Annual dummy variables, controlling for year fixed effects

### 3.3 Model

#### 3.3.1 Main effects model

This study employed balanced panel data and adopted a fixed-effects model based on the results of the Hausman test. To explore the non-linear effect of *SR* on *CIC*, this study introduced *SR*^2^ and constructed the following models:

$$\mathrm{Model}\;(1):\;\begin{array}{l} CIC_{it}=\alpha +\beta_{1}ECO_{it}+\beta_{2}INF_{it}+\beta_{3}IND_{it}+\beta_{4}CON_{it}+\beta_{5}FIN_{it}+\beta_{6}OPEN_{it}+\beta_{7}GOV_{it}+\\ \;\beta_{8}PD_{it}+YEAR+PRO+\varepsilon_{it} \end{array}$$ (1)

$$\mathrm{Model}\;(2):\;\begin{array}{l} CIC_{it}=\alpha +\beta_{1}SR_{it}+\beta_{2}SR_{it}^{2}+\beta_{3}ECO_{it}+\beta_{4}INF_{it}+\beta_{5}IND_{it}+\beta_{6}CON_{it}+\beta_{7}FIN_{it}+\\ \;\beta_{8}OPEN_{it}+\beta_{9}GOV_{it}+\beta_{10}PD_{it}+YEAR+PRO+\varepsilon_{it} \end{array}$$ (2)

Where *α* is constant, *β*_1_~*β*_10_ is the regression coefficient, and *ε*_*it*_ is an error term.

In addition, to examine the non-linear relationship between *SH* and *CIC*, as well as *EM* and *CIC*, two models were established by incorporating *SH*^2^ and *EM*^2^, respectively.

$$\mathrm{Model}\;(3):\;\begin{array}{l} CIC_{it}=\alpha +\beta_{1}SH_{it}+\beta_{2}SH_{it}^{2}+\beta_{3}ECO_{it}+\beta_{4}INF_{it}+\beta_{5}IND_{it}+\beta_{6}CON_{it}+\beta_{7}FIN_{it}+\\ \;\beta_{8}OPEN_{it}+\beta_{9}GOV_{it}+\beta_{10}PD_{it}+YEAR+PRO+\varepsilon_{it} \end{array}$$ (3)

$$\mathrm{Model}\;(4):\;\begin{array}{l} CIC_{it}=\alpha +\beta_{1}EM_{it}+\beta_{2}EM_{it}^{2}+\beta_{3}ECO_{it}+\beta_{4}INF_{it}+\beta_{5}IND_{it}+\beta_{6}CON_{it}+\beta_{7}FIN_{it}+\\ \;\beta_{8}OPEN_{it}+\beta_{9}GOV_{it}+\beta_{10}PD_{it}+YEAR+PRO+\varepsilon_{it} \end{array}$$ (4)

To evaluate the non-linear effects of *SR*, *SH* and *EM* on *OM*, we formulated three models:

$$\mathrm{Model}\;(5):\;\begin{array}{l} OM_{it}=\alpha +\beta_{1}SR_{it}+\beta_{2}SR_{it}^{2}+\beta_{3}ECO_{it}+\beta_{4}INF_{it}+\beta_{5}IND_{it}+\beta_{6}CON_{it}+\beta_{7}FIN_{it}+\\ \;\beta_{8}OPEN_{it}+\beta_{9}GOV_{it}+\beta_{10}PD_{it}+YEAR+PRO+\varepsilon_{it} \end{array}$$ (5)

$$\mathrm{Model}\;(6):\;\begin{array}{l} OM_{it}=\alpha +\beta_{1}SH_{it}+\beta_{2}SH_{it}^{2}+\beta_{3}ECO_{it}+\beta_{4}INF_{it}+\beta_{5}IND_{it}+\beta_{6}CON_{it}+\beta_{7}FIN_{it}+\\ \;\beta_{8}OPEN_{it}+\beta_{9}GOV_{it}+\beta_{10}PD_{it}+YEAR+PRO+\varepsilon_{it} \end{array}$$ (6)

$$\mathrm{Model}\;(7):\;\begin{array}{l} OM_{it}=\alpha +\beta_{1}EM_{it}+\beta_{2}EM_{it}^{2}+\beta_{3}ECO_{it}+\beta_{4}INF_{it}+\beta_{5}IND_{it}+\beta_{6}CON_{it}+\beta_{7}FIN_{it}+\\ \;\beta_{8}OPEN_{it}+\beta_{9}GOV_{it}+\beta_{10}PD_{it}+YEAR+PRO+\varepsilon_{it} \end{array}$$ (7)

To examine the direct relationship between *OM* and *CIC*, this study formulates the following model:

$$\mathrm{Model}\;(8):\;\begin{array}{l} CIC_{it}=\alpha +\beta_{1}OM_{it}+\beta_{2}ECO_{it}+\beta_{3}INF_{it}+\beta_{4}IND_{it}+\beta_{5}CON_{it}+\beta_{6}FIN_{it}+\beta_{7}OPEN_{it}+\\ \;\beta_{8}GOV_{it}+\beta_{9}PD_{it}+YEAR+PRO+\varepsilon_{it} \end{array}$$ (8)

#### 3.3.2 Intermediary effect model

To test whether there is a mediating effect of *OM* in *SR−CIC*, *SH−CIC* and *EM−CIC*, this study formulates three models:

$$\mathrm{Model}\;(9):\;\begin{array}{l} CIC_{it}=\alpha +\beta_{1}SR_{it}+\beta_{2}SR_{it}^{2}+\beta_{3}OM_{it}+\beta_{4}ECO_{it}+\beta_{5}INF_{it}+\beta_{6}IND_{it}+\\ \;\beta_{7}CON_{it}+\beta_{8}FIN_{it}+\beta_{9}OPEN_{it}+\beta_{10}GOV_{it}+\beta_{11}PD_{it}+YEAR+PRO+\varepsilon_{it} \end{array}$$ (9)

$$\mathrm{Model}\;(10):\;\begin{array}{l} CIC_{it}=\alpha +\beta_{1}SH_{it}+\beta_{2}SH_{it}^{2}+\beta_{3}OM_{it}+\beta_{4}ECO_{it}+\beta_{5}INF_{it}+\beta_{6}IND_{it}+\\ \;\beta_{7}CON_{it}+\beta_{8}FIN_{it}+\beta_{9}OPEN_{it}+\beta_{10}GOV_{it}+\beta_{11}PD_{it}+YEAR+PRO+\varepsilon_{it} \end{array}$$ (10)

$$\mathrm{Model}\;(11):\;\begin{array}{l} CIC_{it}=\alpha +\beta_{1}EM_{it}+\beta_{2}EM_{it}^{2}+\beta_{3}OM_{it}+\beta_{4}ECO_{it}+\beta_{5}INF_{it}+\beta_{6}IND_{it}+\\ \;\beta_{7}CON_{it}+\beta_{8}FIN_{it}+\beta_{9}OPEN_{it}+\beta_{10}GOV_{it}+\beta_{11}PD_{it}+YEAR+PRO+\varepsilon_{it} \end{array}$$ (11)

## 4. Empirical analysis and hypothesis testing

### 4.1 Industry competitiveness analysis

Table 7 presents the construction industry’s competitiveness evaluation results for the12 provinces from 2015 to 2020, based on their competitiveness scores. According to the rankings, the development of China’s construction industry has been uneven. In this five-year spans, Jiangsu Province has maintained its first-place ranking in the construction industry competitiveness, which indicates a strong competitive advantage. Conversely, Heilongjiang Province and the Xinjiang Uygur Autonomous Region have ranked poorly, oscillating between 11th and 12th. Moreover, the evaluation results of construction industry competitiveness reveal that the majority of provinces in the eastern region rank in the top five, indicating a well-developed and highly competitive industry. Provinces in the central region’s construction industry competitiveness levels are in the lower middle, with stable ranking fluctuations, which suggests a moderately developed and stable industry. However, most provinces in the western region have poor competitiveness, especially Chongqing and the Xinjiang Uygur Autonomous Region, which rank at the bottom of the 12 provinces. This indicates that the development of the construction industry in Western China is poor and requires improvement.

**Table 7:** Competitive evaluation of the construction industry in twelve provinces, 2015–2020.

Region	Province	Year	CIC	Ranking
Eastern Region	Beijing	2015	0.02	5
		2016	0.35	5
		2017	0.51	5
		2018	0.74	5
		2019	1.53	5
		2020	1.89	5
	Shanghai	2015	-2.05	9
		2016	-1.66	9
		2017	-1.49	9
		2018	-1.4	9
		2019	-1.13	9
		2020	-1.06	9
	Jiangsu	2015	3.42	1
		2016	3.52	1
		2017	3.88	1
		2018	4.66	1
		2019	5.1	1
		2020	5.77	1
	Zhejiang	2015	1.34	2
		2016	1.5	2
		2017	1.77	3
		2018	1.81	4
		2019	1.67	4
		2020	1.94	4
	Shandong	2015	0.66	4
		2016	0.94	4
		2017	1.39	4
		2018	1.89	3
		2019	2.45	3
		2020	3.2	3
	Guangdong	2015	0.73	3
		2016	1.12	3
		2017	1.83	2
		2018	2.65	2
		2019	3.26	2
		2020	3.66	2
Eastern Region	Heilongjiang	2015	-3.29	11
		2016	-3.2	11
		2017	-3.19	11
		2018	-3.15	12
		2019	-3.15	12
		2020	-3.07	12
	Anhui	2015	-1.74	8
		2016	-1.41	8
		2017	-1.18	8
		2018	-0.77	8
		2019	-0.59	8
		2020	-0.13	8
	Hubei	2015	-0.82	6
		2016	-0.71	6
		2017	-0.25	6
		2018	0.47	6
		2019	0.73	7
		2020	0.96	7
Western Region	Chongqing	2015	-2.36	10
		2016	-2.22	10
		2017	-2.1	10
		2018	-2.11	10
		2019	-2.03	10
		2020	-1.93	10
	Sichuan	2015	-1.28	7
		2016	-0.96	7
		2017	-0.38	7
		2018	0.32	7
		2019	0.84	6
		2020	1.08	6
	Xinjiang	2015	-3.44	12
		2016	-3.38	12
		2017	-3.28	12
		2018	-3.02	11
		2019	-2.78	11
		2020	-2.84	11

The construction industry in the eastern regions of China may have achieved better results because its robust economic foundation, which provides ample financial resources and infrastructure for development compared to the central and western regions [58]. Additionally, benefiting from earlier exposure to international markets, these regions have better supportive infrastructure and a more established business environment, enabling them to adopt advanced technologies and best practices from developed nations more readily [59].

### 4.2 Descriptive statistical and correlations

Table 8 shows descriptive statistics of variables. The CIC scores show a maximum of 5.770 to a minimum of -0.3440, and a standard deviation of 2.308, which suggest significant heterogeneity in the construction industry competitiveness across the 12 provinces, with noticeable polarization. Such variation highlights unevenness in the development of China’s construction industry, owing to differing emphases on industry development across the provinces. Similar to the CIC scores, the SR scores rank from a minimum of 7.065 to a maximum of 24.360. The mean value of 19.230 and a standard deviation of 3.498 of SR scores mean social responsibility fulfilment within Chinese construction industry is still in its early stages, while social responsibility awareness persists in the industry across various regions. In contrast to the EM scores, which display a maximum value of 11.120, a minimum value of 0, and a standard deviation of 1.644, the SH scores demonstrate maximum, minimum, and standard deviation values of 18.810, 2.795, and 2.721, respectively. These results imply that the performance level of employee-dimensional social responsibility exhibits less variation compared to that of shareholder-dimensional social responsibility across different regions. Finally, the OM scores reveal a standard deviation of 0.025, with a maximum of 0.058 to a minimum of -0.124, which indicates relatively insignificant variations in the operational status of the construction industry across the provinces.

**Table 8:** Descriptive statistics.

Variables	N	Mean	SD	Min	Max
*CIC*	72	0.000	2.308	-3.440	5.770
*SR*	72	19.230	3.498	7.065	24.360
*SH*	72	12.790	2.721	2.795	18.810
*EM*	72	2.296	1.644	0.000	11.120
*OM*	72	0.023	0.025	-0.124	0.058
*ECO*	72	11.170	0.428	10.400	12.010
*INF*	72	5.850	6.786	-25.200	16.500
*IND*	72	0.311	0.073	0.118	0.419
*CON*	72	0.065	0.028	0.018	0.120
*FIN*	72	0.088	0.040	0.052	0.196
*OPEN*	72	0.594	0.481	0.057	1.829
*GOV*	72	0.221	0.083	0.124	0.430
*PD*	72	2723	1121	1136	5515

Table 9 shows Pearson correlation between the principal variables. The highest correlation among the variables is 0.776, which is less than 0.8. Thus, it indicates a low possibility of multicollinearity.

**Table 9:** Correlation statistics.

	*CIC*	*SR*	*SH*	*EM*	*OM*
*CIC*	1				
*SR*	0.319***	1			
*SH*	0.414***	0.748***	1		
*EM*	0.075	0.108	-0.022	1	
*OM*	0.428***	0.648***	0.776***	-0.093	1

Note. The p-values are indicated with asterisks. The correlation is significant at the 1%, 5%, and 10% levels. The variables are defined as described above.

### 4.3 Main effect analysis

Table 10 presents the results of the main-effects test. The results of Model (1) are shown in Column 1, which suggests that economic development, industrial development, construction industry development, financial industry development, openness to the outside world, and population density are all significant contributors to the improvement in industry competitiveness. Notably, the level of infrastructure construction was negatively correlated with industry competitiveness.

**Table 10:** Results of main effects test.

Variables	Model (1)	Model (2)	Model (3)	Model (4)	Model (5)	Model (6)	Model (7)
*SR*		0.237**			0.014***		
		(0.098)			(0.004)		
*SR* ^*2*^		-0.007**			-0.0003**		
		(0.003)			(0.000)		
*SH*			0.233***			0.020***	
			(0.085)			(0.003)	
*SH* ^*2*^			-0.010***			-0.001***	
			(0.004)			(0.000)	
*EM*				-0.402***			-0.014*
				(0.114)			(0.007)
*EM* ^*2*^				0.032***			0.001
				(0.009)			(0.001)
*ECO*	3.398*	4.963**	3.457*	4.402**	-0.062	-0.183**	-0.059
	(1.889)	(1.965)	(1.852)	(1.739)	(0.083)	(0.071)	(0.108)
*INF*	-0.025***	-0.020**	-0.016**	-0.028***	0.000	0.001*	0.000
	(0.007)	(0.007)	(0.008)	(0.007)	(0.000)	(0.000)	(0.000)
*IND*	15.454***	12.494**	10.218*	18.416***	-0.068	-0.334	0.169
	(5.428)	(5.364)	(5.475)	(4.959)	(0.227)	(0.210)	(0.308)
*CON*	46.725***	36.798**	30.066*	42.572***	-0.348	-1.258**	-0.514
	(15.407)	(15.536)	(15.843)	(14.014)	(0.659)	(0.608)	(0.869)
*FIN*	28.281**	16.874	17.186	27.707**	0.270	-0.268	0.858
	(13.304)	(13.639)	(13.282)	(12.02)	(0.578)	(0.510)	(0.746)
*OPEN*	1.596***	1.594***	1.548***	1.788***	-0.012	-0.016	-0.006
	(0.335)	(0.322)	(0.317)	(0.306)	(0.014)	(0.012)	(0.019)
*GOV*	4.436	3.888	4.426	1.129	-0.470*	-0.615***	-0.735**
	(6.262)	(6.043)	(-5.927)	(5.806)	(0.256)	(0.228)	(0.360)
*PD*	0.001***	0.001***	-0.010***	0.001***	0.000	0.000	0.000
	(0.000)	(0.000)	(0.004)	(0.000)	(0.000)	(0.000)	(0.000)
*YEAR*	*control*	*control*	*control*	*control*	*control*	*control*	*control*
*PRO*	*control*	*control*	*control*	*control*	*control*	*control*	*control*
*_cons*	-52.389**	-68.361***	-50.166**	-63.176***	0.702	2.274**	0.741
	(23.120)	(23.486)	(22.504)	(21.234)	(0.996)	(0.864)	(1.317)
*R* ^*2*^	0.845	0.862	0.867	0.879	0.701	0.763	0.438
*Adj-R* ^*2*^	0.765	0.783	0.790	0.809	0.528	0.626	0.114

Note. The p-values are indicated with asterisks. The correlation is significant at the1%, 5%, and 10% levels. The variables are defined as described above.

Models (2), (3), and (4) were designed based on Model (1), which introduced the primary and secondary terms of the three independent variables into the regression models. The results are shown in Columns 2, 3, and 4.

The results confirm the inverted U-shaped relationship between SR and CIC (*β*_2_ = −0.007 and *p*<0.05, *β*_1_ = 0.237 and *p*<0.05), which supports H1. Active social responsibility fulfilment in the construction industry can improve overall competitiveness; however, exceeding a certain threshold may hurt competitiveness and result in the ‘too much is too little’ phenomenon. This is consistent with the findings of Guo and Lu [30]. We argued that excessive social responsibility investment sacrifices the rights and interests of some stakeholders and reduces their support for the organisation, thus hindering the improvement of competitiveness. Moreover, bidding is an important link in the production process of the construction industry. Excessive social responsibility can lead to higher prices and lower bidding competitiveness.

According to the results in Column 3, there is the ‘inverted U-shaped’ relationship exists between SH and CIC (*β*_2_ = −0.010 and *p*<0.01, *β*_1_ = 0.233 and *p*<0.05), supporting H1a. This may be because the fulfilment of shareholder social responsibility is a type of investment. For China’s construction industry, investment rationality is crucial for the improvement of industry competitiveness. Reasonable investments can help accumulate external social capital and enhance competitiveness. Conversely, excessive investment can displace other investments and negative effects on the industry’s competitiveness, which results in ‘not being able to make ends meet’.

Column 4 shows the U-shaped relationship between EM and CIC (*β*_2_ = 0.032 and *p*<0.01, *β*_1_ = −0.402 and *p*<0.01), supporting H1b. This indicates that employee-dimensional social responsibility needs a certain degree of accumulation to gradually and significantly improve industry competitiveness. Employee-dimensional social responsibility fulfilment is an important way to improve employee satisfaction, especially social responsibility activities such as increasing employees’ welfare and improving their working conditions. These social responsibility activities can not only stimulate the enthusiasm of employees but also promote improved quality of human resources and increased competitive advantages for the organization [60]. The construction industry is labour intensive, and relies on good human resources to remain competitive. However, in the initial stages, scarce resources such as capital, facilities, and human resources necessary for competitiveness enhancement may be occupied, which leads to a failure to use potential benefits rationally and affects competitiveness enhancement [33]. With mature employee-dimensional social responsibility, potential benefits transform into tangible outcomes. On the one hand, an organisation can form a good reputation to enhance its attractiveness to potential employees [61]. On the other hand, the potential benefits of employee-dimensional social responsibility fulfilment, such as the improvement of employees’ work literacy and potential, need to be accumulated for a period of time to be transformed into employees’ comprehensive abilities. These help improve the quality of human resources, thereby enhancing the value creation ability of the organisation and ultimately improving industry competitiveness.

Model (8) is based on Model (1) and introduces OM into the regression model. According to the results in Table 11, a significantly positive relationship between OM and CIC is confirmed (*β*_1_ = 5.162, *p*<0.05), which supports H3. This finding may be attributed to the fact that strong operations management is not only closely related to the operation and development of construction enterprises but also the basis for enhancing construction industry competitiveness. On one hand, a higher level of OM can help enterprises conduct various business activities. On the other hand, favourable OM enables enterprises to operate efficiently and enhance their ability to manage risks and seize opportunities, which will help enterprises maintain a leading position in market competition. Enterprises are important components of an industry. Therefore, improving the competitiveness of construction enterprises can improve the overall value of the construction industry, gain a higher market share for the construction industry, and improve the industry’s competitive advantage.

**Table 11:** Intermediary effect results.

Variables	Model (8)	Model (9)	Model (10)
*SR*		0.153	
		(0.108)	
*SR* ^*2*^		-0.005*	
		(0.003)	
*SH*			0.146
			(0.115)
*SH* ^*2*^			-0.007
			(0.004)
*EM*			
		
*EM* ^*2*^			
		
*OM*	5.162**	6.028*	4.335
	(2.448)	(3.437)	(3.872)
*ECO*	3.760**	5.340***	4.249**
	(1.832)	(1.934)	(1.978)
*INF*	-0.023***	-0.020***	-0.018**
	(0.007)	(0.007)	(0.008)
*IND*	15.036***	12.907**	11.664**
	(5.243)	(5.250)	(5.610)
*CON*	48.069***	38.899**	35.520**
	(14.885)	(15.237)	(16.533)
*FIN*	23.341*	15.246	18.346
	(13.053)	(13.367)	(13.285)
*OPEN*	1.653***	1.670***	1.618***
	(0.324)	(0.318)	(0.322)
*GOV*	7.281	6.723	7.089
	(6.193)	(6.126)	(6.371)
*PD*	0.001***	0.001***	0.001**
	(0.000)	(0.000)	(0.000)
*YEAR*	*control*	*control*	*control*
*PRO*	*control*	*control*	*control*
*_cons*	-56.630**	-72.594***	-60.025**
	(22.406)	(23.089)	(24.106)
*R* ^*2*^	0.858	0.871	0.871
*Adj- R* ^*2*^	0.781	0.792	0.791

Note. The p-values are indicated with asterisks. The correlation is significant at the 1%, 5%, and 10% levels. The variables are defined as described above.

Models (5), (6), and (7) were used to examine the relationship between the three social responsibilities and enterprise operations management, and the results are shown in Columns 5, 6, and 7 of Table 10. The results in Column 5 support the inverted U-shaped relationship between SR and OM (*β*_2_ = −0.0003 and *p*<0.05, *β*_1_ = 0.014 and *p*<0.01), thus verifying H2. Excessive social responsibility fulfilment may result in investing more resources to fulfil them than the benefits they generate, ultimately creating a debt burden that hinders industry operations management. Therefore, the impact of the SR on OM tended to first increase and then decrease, which is consistent with the results of Sang et al. [44]. They argued that the relationship between stakeholders and construction projects is one-time in nature; the limited resources of stakeholders cannot provide lasting support to the organisation, and social responsibility fulfilment beyond a certain threshold may negatively affect operation management.

Based on the results shown in Table 10, an inverted U-shaped relationship between SH and OM was confirmed (*β*_2_ = −0.001 and *p*<0.01, *β*_1_ = 0.020 and *p*<0.01), which supports H2a. This result indicates that excessive fulfilment of shareholder-dimensional social responsibility is not conducive to improving the operation and management levels of construction enterprises. One possible explanation for these results is that shareholder-dimensional social responsibility fulfilment encourages shareholders to invest resources in operational activities, thereby contributing to an enterprise’s economic development. However, most large construction companies in China are state-owned. The government controls the behaviour of enterprises and formulates policies that are in the best interests of the country and multiple-stakeholders [62]. Therefore, shareholders’ investment decision-making behaviour is subject to certain constraints. In this context, the positive response of shareholders to the shareholder-dimensional social responsibility behaviour of construction enterprises is also limited, and excessive investments may not receive equivalent shareholder resource feedback. In addition, excessive shareholder-dimensional social responsibility activities cause other interest groups to dislike corporate behaviour. These negative effects affect the normal operational activities of enterprises, hindering the improvement of enterprise operation management.

According to Table 10, EM^2^ is not significantly correlated with OM; thus, H2b does not hold. Moreover, the stepwise test of the mediating effect reveals that if hypothesis H2b does not hold, hypothesis H4b does not hold. The reason for this result may be that social responsibility fulfilment can increase employee satisfaction and turnover, especially employee-dimensional social responsibility fulfilment, such as organizational commitment [63]. Organizational commitment can enhance employee performance and well-being [64] by improving employees’ attitudes toward and perceptions of work [65], but its positive impact may depend on employees’ cultural values [66]. Employee perceptions and organizational commitment are positively correlated only if the cultural values of the enterprise and its employees are consistent. Chinese construction firms that contract with international projects must recruit a certain percentage of local employees. Thus, Chinese and local employees, along with management and technical experts from other countries, were present at the project site. As a result, the Chinese construction industry has a wide range and large number of employees [67] and is characterised by international and geographical diversity [68]. Given this background, it is difficult to unify the cultural values among enterprises. Employee-dimensional social responsibility may not improve employees’ organizational perceptions or work efficiency, and thus may have little impact on enterprises’ OM, let alone indirectly improve the competitiveness by improving the enterprise’s OM.

### 4.4 Mediating effects of industry operation management

Table 11 reports the results of the mediating effect of OM on the relationship between SR and CIC, and the relationship between SH and CIC. The analysis follows the fixer-effect regression models (9) and (10).

In Column 2, the incorporation of the mediating variable OM into the main effects model reveals a significantly negative relationship between SR^2^ and CIC (*β*_2_ = −0.005, *p*<0.1). Moreover, the absolute value of the correlation coefficient decreases, indicating that OM serves as a partial mediator in the relationship between SR and CIC. This result is consistent with H4. Based on resource dependency theory, social responsibility fulfilment is vital for organisations to obtain key resources from stakeholders, which can reduce the risks associated with resource acquisition and secure high-quality resources needed for their survival [29]. Consequently, it creates an enabling environment for an enterprise’s operational activities and generates financial income. In the construction industry, social responsibility activities are typically conducted through employee skills training, safety education, and infrastructure construction at project sites, which incur additional costs [30] such as facility and management costs. In the early stages of social responsibility fulfilment, the revenue generated can cover its initial costs and effectively enhance enterprise competitiveness by improving operation management, which helps the enterprise obtain competitive advantages. However, as the marginal benefit of social responsibility decreases, exceeding a certain threshold may lead to income being unable to compensate for the costs. Thus, excessive social responsibility fulfilment has negative effects on the improvement of an enterprise’s OM level and leads to a decline in its competitiveness. With the decline in enterprise competitiveness, the industry will also be at a disadvantage in market competition, which is not conducive to the industry occupying a favourable market share, thus casuing negative effects on industry competitiveness improvement. Therefore, optimal levels of social responsibility fulfilment are essential for maximising benefits and gaining competitive advantage through improved OM, where neither too little nor too much fulfilment is optimal.

Column 3 of Table 11 presents the results of the regression analysis of Model (10). The results indicated that SH^2^, OM, and CIC were not significantly correlated, suggesting that OM did not mediate the relationship between SH and CIC. Thus, H4a was verified as untenable. Shareholder-dimension social responsibility is considered an economic responsibility vital for the survival and growth of construction enterprises. Nevertheless, with China’s construction industry transitioning toward high-quality development, social responsibility is developing in a multi-dimensional manner. Consequently, single-dimensional social responsibility fulfilment is insufficient to meet market demands, which results in fewer positive effects on enterprises. Moreover, SH may not significantly enhance CIC induced by OM. To achieve positive effects, it is crucial to integrate and fulfil all the dimensions of social responsibility.

### 4.5 Robustness tests

#### Replace key variables

This study investigates the potential impact of variable measurement errors on the empirical analysis results. To guarantee the reliability of the outcomes, we performed a robustness test by substituting the mediating variable with the mean value of return on net assets (ROE) to measure industry operations management (OM_2), followed by re-executing the regression analysis. The test results presented in Table 12 validate the consistency of the prior findings, confirming the robustness of the previous empirical analysis outcomes.

**Table 12:** Substitution of intermediary variables.

Variables	Model (5)	Model (6)	Model (8)	Model (9)
*SR*	0.055***			0.122
	(0.017)			(0.104)
*SR* ^*2*^	-0.001**			-0.004
	(0.000)			(0.003)
*SH*		0.075***		
		(0.012)		
*SH* ^*2*^		-0.002***		
		(0.001)		
*OM_2*			1.961***	2.082**
			(0.669)	(0.843)
*ECO*	-0.465	-0.926***	4.573**	5.932***
	(0.329)	(0.266)	(1.798)	(1.903)
*INF*	-0.000	0.001	-0.021***	-0.019**
	(0.001)	(0.001)	(0.007)	(0.007)
*IND*	0.066	-0.909	14.112***	12.356**
	(0.899)	(0.787)	(5.057)	(5.084)
*CON*	-0.775	-3.948*	46.371***	38.412**
	(2.604)	(2.276)	(14.295)	(14.739)
*FIN*	0.492	-1.22	22.056*	15.85
	(2.286)	(1.908)	(12.525)	(12.932)
*OPEN*	-0.041	-0.055	1.668***	1.679***
	(0.054)	(-0.046)	(0.311)	(0.307)
*GOV*	-1.881*	-2.247**	8.476	7.805
	(1.013)	(0.851)	(5.971)	(5.943)
*PD*	-0.000	0.000	0.001***	0.001***
	(0.000)	(0.000)	(0.000)	(0.000)
*YEAR*	*control*	*control*	*control*	*control*
*PRO*	*control*	*control*	*control*	*control*
*_cons*	5.083	10.985***	-65.251***	-78.946***
	(3.936)	(3.233)	(21.895)	(22.666)
*R* ^*2*^	0.594	0.711	0.869	0.879
*Adj- R* ^*2*^	0.359	0.545	0.798	0.805

Note. The p-values are indicated with asterisks. The correlation is significant at the 1%, 5%, and 10% levels. The variables are defined as described above.

#### Endogeneity test

Considering the potential presence of endogeneity issues, including reverse causality and measurement error, we use two-stage least squares (2SLS) regressions with instrumental variables (IVs) to address the potential endogeneity concerns. In this study, the mean SR values (SR_AVG) and SR with a one-period lag (L.SR) were used as the two IVs for SR. Table 13 presents the results of the IV-SLS regression. This further supports the inverted U-type relationship between the SR and CIC, which is consistent with previous results. Therefore, it can be shown that there is no serious endogeneity problem in this study and the results are robust.

**Table 13:** Tool variables.

IV	*SR_AVG*	*L*.*SR*
*SR*	1.134*	0.806*
	(0.663)	(0.417)
*SR* ^*2*^	-0.034*	-0.026**
	(0.019)	(0.011)
*Controls*	*control*	*control*
*YEAR*	*control*	*control*
*PRO*	*control*	*control*
*R* ^*2*^	0.878	0.879
*Adj- R* ^*2*^	0.845	0.841

Note. The p-values are indicated with asterisks. The correlation is significant at the 1%, 5%, and 10% levels. The variables are defined as described above.

In addition, this study considers that there may be an inverse causal relationship between SR and CIC. To further solve the endogeneity issue, explanatory variables are lagged by one period (L.CIC) for the regression analysis. The results in Table 14 confirm the presence of an inverted U-shaped relationship between SR and CIC as well as between SH and CIC. The results in Column 3 reveal a U-shaped relationship between EM and CIC. Thus, the endogeneity problem of this study is once again proven to be insignificant, and the analysis results are robust.

**Table 14:** Lagged one-period dependent variable regression results.

Variables	Model (2)	Model (3)	Model (4)
*SR*	0.193*		
	(0.100)		
*SR* ^*2*^	-0.005*		
	(0.003)		
*SH*		0.253***	
		(0.078)	
*SH* ^*2*^		-0.010***	
		(0.003)	
*EM*			-0.288*
			(0.149)
*EM* ^*2*^			0.023*
			(0.012)
*ECO*	4.229	3.080	5.684**
	(2.611)	(2.285)	(2.453)
*INF*	-0.022**	-0.019**	-0.028***
	(0.008)	(0.008)	(0.008)
*IND*	17.753***	16.387***	19.725***
	(6.254)	(5.980)	(6.162)
*CON*	57.668***	47.101***	62.689***
	(17.372)	(16.974)	(17.076)
*FIN*	15.408	14.071	33.912**
	(16.233)	(14.669)	(14.820)
*OPEN*	0.776**	0.780**	0.984***
	(0.339)	(0.312)	(0.346)
*GOV*	11.829*	12.136**	10.518*
	(5.943)	(5.501)	(6.160)
*PD*	0.001**	0.001***	0.001***
	(0.000)	(0.000)	(0.000)
*YEAR*	*control*	*control*	*control*
*PRO*	*control*	*control*	*control*
*_cons*	-64.937**	-50.901*	-82.180***
	(30.791)	(27.237)	(29.246)
*R* ^*2*^	0.854	0.874	0.851
*Adj-R* ^*2*^	0.747	0.781	0.741

Note. The p-values are indicated with asterisks. The correlation is significant at the 1%, 5%, and 10% levels. The variables are defined as described above.

## 5. Discussion

Although the academic community has studied the relationship between social responsibility fulfilment and competitiveness extensively, existing research has focused mostly on the linear relationship between the two [69–71] on the enterprise level [72] with relatively little research on the relationship between the two from the industry perspective. Therefore, by constructing a model mediated by OM, this study explores whether and how the fulfilment of social responsibility in the construction industry improves competitiveness. Based on relevant data on the construction industry in 12 provinces in China from 2015 to 2020, this study empirically tested the theoretical hypotheses. The main findings and implications of this study are as follows.

Our research finds that there is a non-linear relationship between SR and CIC, and the correlation curve between the two is an inverted ‘U’ shape, thus supporting hypothesis H1. By meeting the expectations and demands of various interest groups, social responsibility activities can obtain key resources such as funds, reputation, and image for organisations [73]. This can effectively improve the competitiveness of the construction industry in terms of value creation and ability. However, excessive social responsibility may inhibit improvements in industrial competitiveness. According to expectation breach theory, stakeholders compare the actual performance of an organisation with their subjective expectations, and once the actual performance is worse than expected, an expectation breach will occur [74]. In the construction industry, excessive fulfilment of social responsibility may raise the expectations of stakeholders, thus strengthening the expectation-breach effect. However, this effect causes negative emotions, which have a negative impact on the organisation. This reduces social responsibility performance and wastes resources, thus inhibiting competitiveness. Excessive social responsibility fulfilment has negative effects, which is consistent with the results of Guo et al. [30]. This study also expands on the research of Guo et al. [30] and considers the impact of different dimensions of social responsibility fulfilment (shareholders and employees) on industry competitiveness. The empirical results show that there is an inverted ‘U’ type interaction between SH and CIC and a positive ‘U’ type interaction between EM and CIC, respectively, which supports hypotheses H1a and H1b. The emergence of these differentiated results may be related to the current situation in China’s construction industry and the development of social responsibility. In the early stage of social responsibility construction, the construction industry focused only on ‘shareholder primacy’, which led to an alienation from the principles of social responsibility via, e.g., ‘greenwashing’ behaviour of false publicity and the ‘decoupling’ behaviour of only saying and not doing [75]. With the emergence of these problems, employees, the public, and other interest groups are repulsed by this idea and produce opposing emotions in shareholders, leading to contradictions and conflicts among various interest groups. In this context, the excessive fulfilment of shareholder social responsibility in the construction industry will not only affect the development of other business activities due to the crowding of internal resources but also worsen relationships with employees, the public, and other groups. This will trigger negative external effects on the organisation, which is not conducive to improving competitiveness. China’s construction industry is still in a critical period of transformation toward high-quality development; thus, improving the quality of human resources is crucial [76]. Therefore, the academic community and industry should pay more attention to effectively fulfilling their social responsibilities to employees by improving the quality of skill training and optimising the working environment to improve their work literacy and stimulate their work potential and enthusiasm. However, this potential benefit may not play a positive role in the early stages of the implementation of employee social responsibility and may even have negative effects. Only after a period of accumulation can they be externalised into the comprehensive ability of employees, thus creating considerable value for the organisation and ultimately boosting its competitiveness.

Both SR and SH have an inverted ‘U’ shaped impact on OM, which supports hypotheses H2 and H2a. This finding is supported by existing research [44], which emphasizes the negative effects of excessive social responsibility on corporate operations and management. This may be related to the market environment of China’s construction industry. Since the policy of ‘housing without speculation’ was put forward, China’s real estate industry has been in a downturn. The construction industry is an upstream industry, and its development is not optimistic. In this depressed market environment, investment rationality is particularly important. Excessive investment in social responsibility increases corporations’ operational risk, affects the normal development of operational activities, and hinders the improvement of operations management levels. In addition, excessive social responsibility activities send signals of idle resources to the outside world, thus establishing a negative image of improper enterprise management [56]. Specifically, excessive shareholder social responsibility activities hinder the diversified and balanced development of other dimensions of social responsibility, thus affecting stakeholders’ perceptions of CSR behaviour. They believe that corporate social responsibility behaviour focuses only on the interests of shareholders and ignores the welfare of other interest groups that have a certain symbolic value. On the contrary, it will establish a negative image of the enterprise as ‘not responsible’, and reduce the enthusiasm of other interest groups to participate in the operation activities, and ultimately hinder the improvement of the operation and management of the enterprise. However, EM did not significantly affect OM, which does not support H2b. In contrasts the results of Cavazotte and Chang [77], who believe that employee social responsibility inputs such as employee education and environmental improvement can play a positive role in the process of enterprise operation and management. This difference may be related to the diversity of types of employees in China’s construction industry. Employees in China’s construction industry come from a wide range of locations and have different cultural cognition and educational backgrounds. Such individual differentiation leads to different cognitions of social responsibility among employees, thus affecting their views on organizational social responsibility behaviors [78]. In addition, different employee groups may differ significantly in social responsibility demands and expectations owing to different cultural values and interest needs. These factors may lead to significant conflicts regarding social responsibility concepts among various employee groups. In this context, the employee social responsibility activities of construction enterprises may not fully meet the wishes of some employees; thus, they think that corporate behaviour has strong symbolism and may even question the cultural values of the enterprises. In particular, the form and content of employee-dimensional social responsibility activities in China’s construction industry are relatively simple at this stage and cannot meet the practical needs of various employee groups in a balanced manner. All these factors will affect the formation of consistent cultural values and long-term goals within the enterprise, which is not conducive to mobilising employees’ work enthusiasm and improving their participation in operational activities. Therefore, at this stage, the fulfilment of employee social responsibility in the construction industry cannot have a significant impact on the improvement in the OM level.

In addition, we made valuable discoveries. OM has a partial mediating effect on the relationship between SR and CIC, which supports H3 and H4. In other words, the fulfilment of social responsibility can also promote the improvement of the competitiveness of the construction industry by improving enterprise operations and management. The construction industry has a wide social impact, and its businesses involve many social groups. By reasonably conducting social responsibility activities for different stakeholders, the construction industry can enhance the adhesion and interaction between these stakeholders and enterprises and encourage them to establish a good relationship between dependence and cooperation with various social groups [67]. In this social responsibility interaction, construction enterprises can obtain the key resources needed for the survival and development of the organisation from stakeholders, thereby reducing the risk of resource acquisition. In addition, the synergistic effect between construction enterprises and various interest groups can realise the integration of resources and capabilities and significantly enhance their value-creation ability. This will help improve the economic performance of enterprises, thus creating a good economic environment for their operations. Corporations can invest more funds in high-return and high-yield operational activities, create higher comprehensive benefits, improve the level of operation and management of enterprises, and help corporations obtain competitive advantages, thus triggering a positive impact on the competitiveness of the construction industry. However, once the level of social responsibility fulfilment in the construction industry exceeds a certain threshold, there may be negative effects. The development of social responsibility activities in the construction industry is often accompanied by large investments in resources. Excessive investment crowds out the limited resources of the enterprise, thus affecting the normal operational activities of the enterprise, improving the operational risk of the corporation, and leading to a decline in the operation management level of the enterprise. However, excessive social responsibility activities may increase stakeholders’ expectations of social responsibility excessively. Once the distribution of corporates’ social responsibility input is biased, it will cause negative emotions in some social groups and even lead to the deterioration of the relationship between enterprises and stakeholders. These negative effects not only affect the development of enterprise operational activities but also bring greater operational risks, which is not conducive to the formation of enterprise competitive advantage and ultimately hinders the improvement of industry competitiveness.

## 6. Conclusion

This study calculated the fulfilment level of social responsibility of the construction industry in 12 provinces of China from 2015 to 2020 using the time-series global PCA method and analysed the impact mechanism of social responsibility fulfilment on construction industry competitiveness by constructing a multiple regression model and an intermediary effect model, which enriches the research results in the realm of social responsibility and broadens the research scope. Through empirical analysis, this study obtained valuable findings. Currently, the development of the construction industry in China’s provinces and cities is not balanced, and the competitiveness of the industry has room for improvement. There is an inverted U-shaped interactive relationship between SR and CIC, and a different interactive relationship between the fulfilment level of social responsibility and industry competitiveness in a single dimension (shareholders and employees). However, the implementation of employee-dimension social responsibility has certain limitations in terms of improving the operational management level. In addition, the construction industry should also pay attention to the intermediary mechanism of ‘SR—OM—CIC’. The research results have important implications for promoting high-quality social responsibility in China’s construction industry and enhancing industry competitiveness.

First, construction enterprises should promote the diversified development of social responsibility according to the characteristics of the different stakeholders. This is because social responsibility fulfilment is beneficial for the improvement of the construction industry competitiveness; however, it has limitations. In particular, the impact of the different dimensions of social responsibility fulfilment (shareholders and employees) on industry competitiveness is quite different. Therefore, construction enterprises should not only comprehensively promote the high-quality construction of all dimensions of social responsibility but should also formulate targeted strategies for fulfilling social responsibility according to the differentiated impact of different dimensions of social responsibility on competitiveness.

Second, construction enterprises should strengthen the balanced construction of social responsibility through diversification. This is because, although social responsibility fulfilment can effectively improve the operational and management performance of enterprises, it is often difficult to work if the fulfilment of social responsibility is overreliant on a single dimension. For example, homogenised employee social responsibility activities may be influenced by personal cultural values and interest demands but cannot have a significant impact on enterprise operation and management. This suggests that we must establish a balanced relationship between all dimensions of social responsibility. On the one hand, relevant government departments and construction industry associations can encourage enterprises to eliminate the habit of over-relying on a certain dimension of social responsibility by promulgating and adjusting corresponding policies, standards, and measures; attach importance to the balanced construction of social responsibility in all aspects; and allocate resources reasonably among various dimensions of social responsibility. On the other hand, construction enterprises should also give full weight to their own expertise and formulate social strategies with situational adaptability according to the social responsibility needs and expectations of different subjects. Moreover, this type of equilibrium is relative and not the absolute average of the resource input. Enterprises should combine their own characteristics, engineering characteristics, market environment, and operational strategy; consider other dimensions of social responsibility based on key social responsibilities; and improve the comprehensive effectiveness of social responsibility construction with high-quality social responsibility as a whole.

Third, construction enterprises should promote the coordination of social responsibility fulfilment based on balance. Construction enterprises should optimise how they perform social responsibility to achieve full cooperation between the implementation of multi-dimensional social responsibility, pay attention to play, enhance the synergistic effect between the construction of multi-dimensional social responsibility, produce a multiplier effect on the implementation effect, and make the implementation of social responsibility highly adaptable to the sustainable development strategy of enterprises. In particular, it is necessary to clarify the cooperative development logic of multi-dimensional social responsibility and formulate a cooperative implementation strategy for social responsibility.

Finally, according to the intermediary mechanism of ‘social responsibility fulfilment—enterprise operation management—industry competitiveness’ found in this study, it is suggested that enterprise managers should not only insist on improving the quality of social responsibility fulfilment but also strengthen the improvement of the operation and management level of construction enterprises. Moreover, enterprise managers should take this as a starting point to promote the deep integration of social responsibility fulfilment and industry competitiveness improvement. Relevant strategy makers can also refer to the results of this study to formulate new social responsibility implementation and sustainable competitiveness development strategies to strengthen the synergistic effects among social responsibility effectiveness, OM, and industry competitiveness.

Although this study achieved its desired goal and reached some interesting conclusions, it does possess some limitations, which provide new perspectives for follow-up research. The sample selection in this study was limited to China, which is an emerging economy, and may not fully reflect the overall situation of the construction industry in all developing countries. Future studies can expand the sample scope and add relevant data on the construction industry in other developing countries to enhance the representativeness and universality of the research results. The analysis method of this study needs to be optimised, and only quantitative analysis is used to explore the influence of the construction industry’s social responsibility on industry competitiveness. We suggest that future studies use a combination of qualitative and quantitative methods to further explore the possible interaction mechanisms between the two. Finally, in follow-up research, the influence of different internal and external factors on the non-linear relationship between social responsibility and competitiveness, such as employee perception, organizational culture ethics, and media attention, is further discussed to enrich the relevant theories in this field.
